# Supplementary figures and images for: An intra-family conserved high-order RNA structure within the M ORF is important for arterivirus subgenomic RNA accumulation and infectious virus production
Source: J Virol. 2025 Apr 7;99(5):e02167-24. doi: 10.1128/jvi.02167-24 (PMC7617654; doi:10.1128/jvi.02167-24)

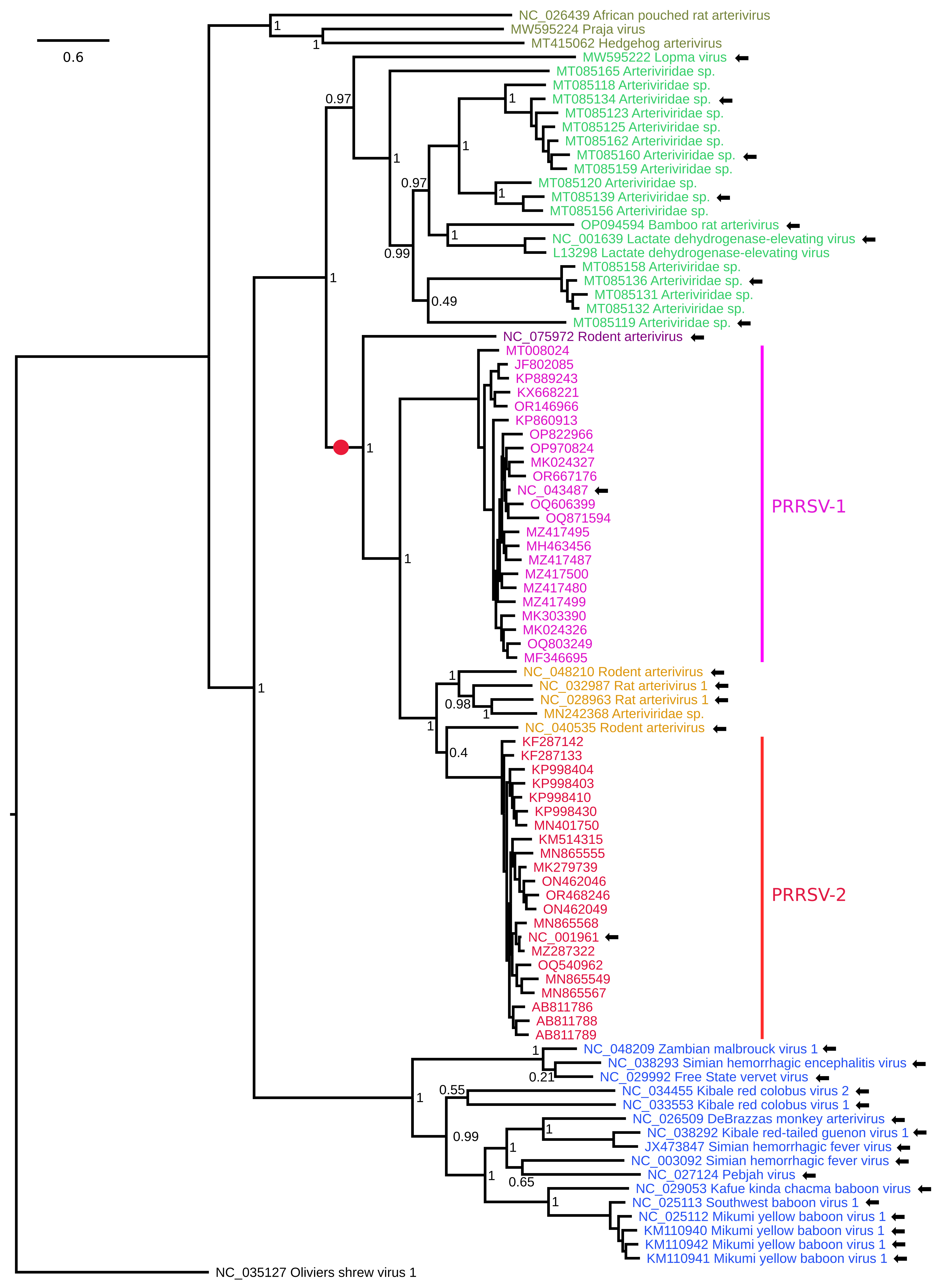

Supplement: Figure S1 — Arterivirus phylogenetic tree. [file jvi.02167-24-s0001.tif]

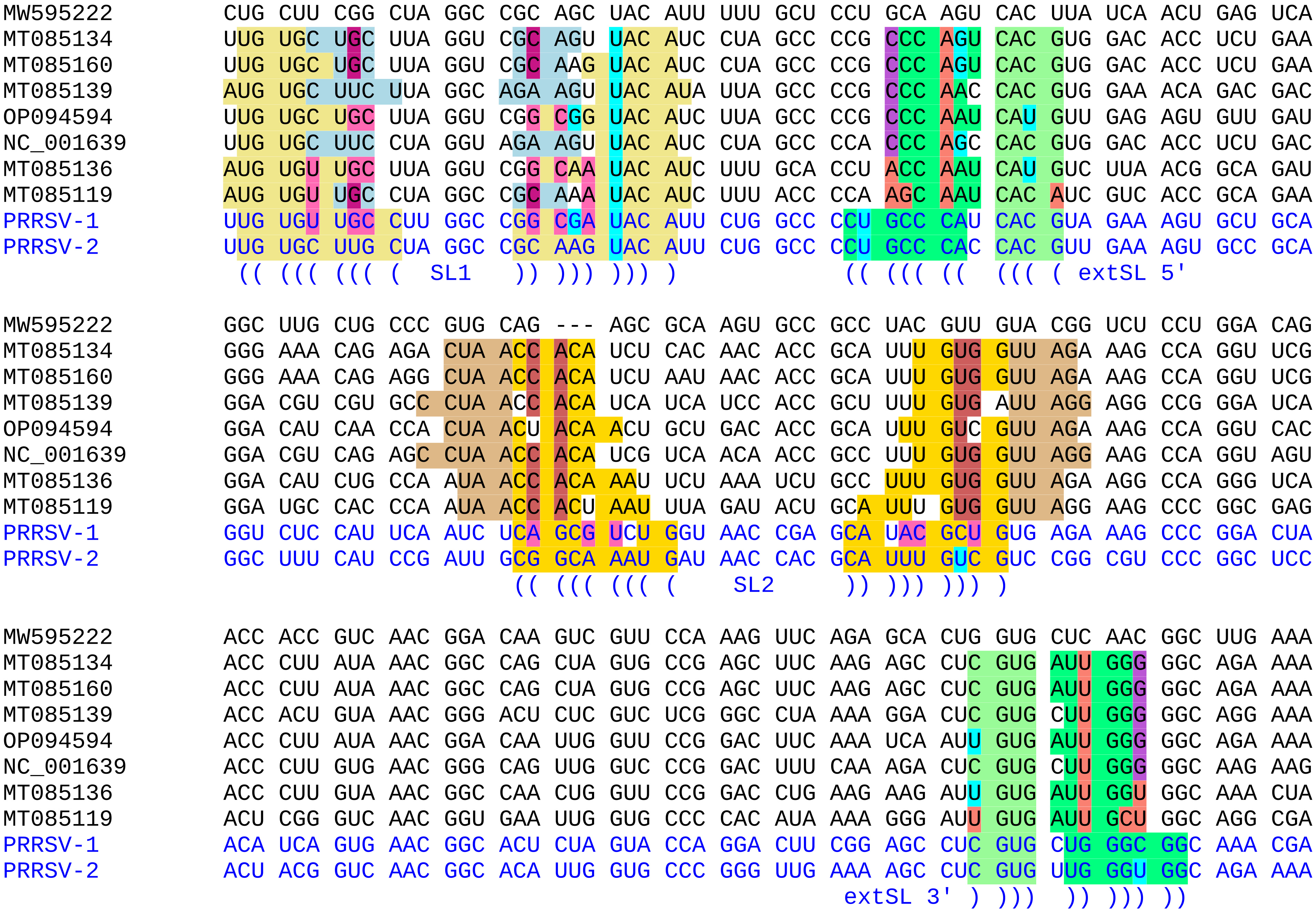

Supplement: Figure S2 — Potential conservation of SL1, SL2, and extSL in lactate dehydrogenase-elevating virus (genus Gammaarterivirus) and relatives, but not in Lopma virus (MW595222). [file jvi.02167-24-s0002.tif]

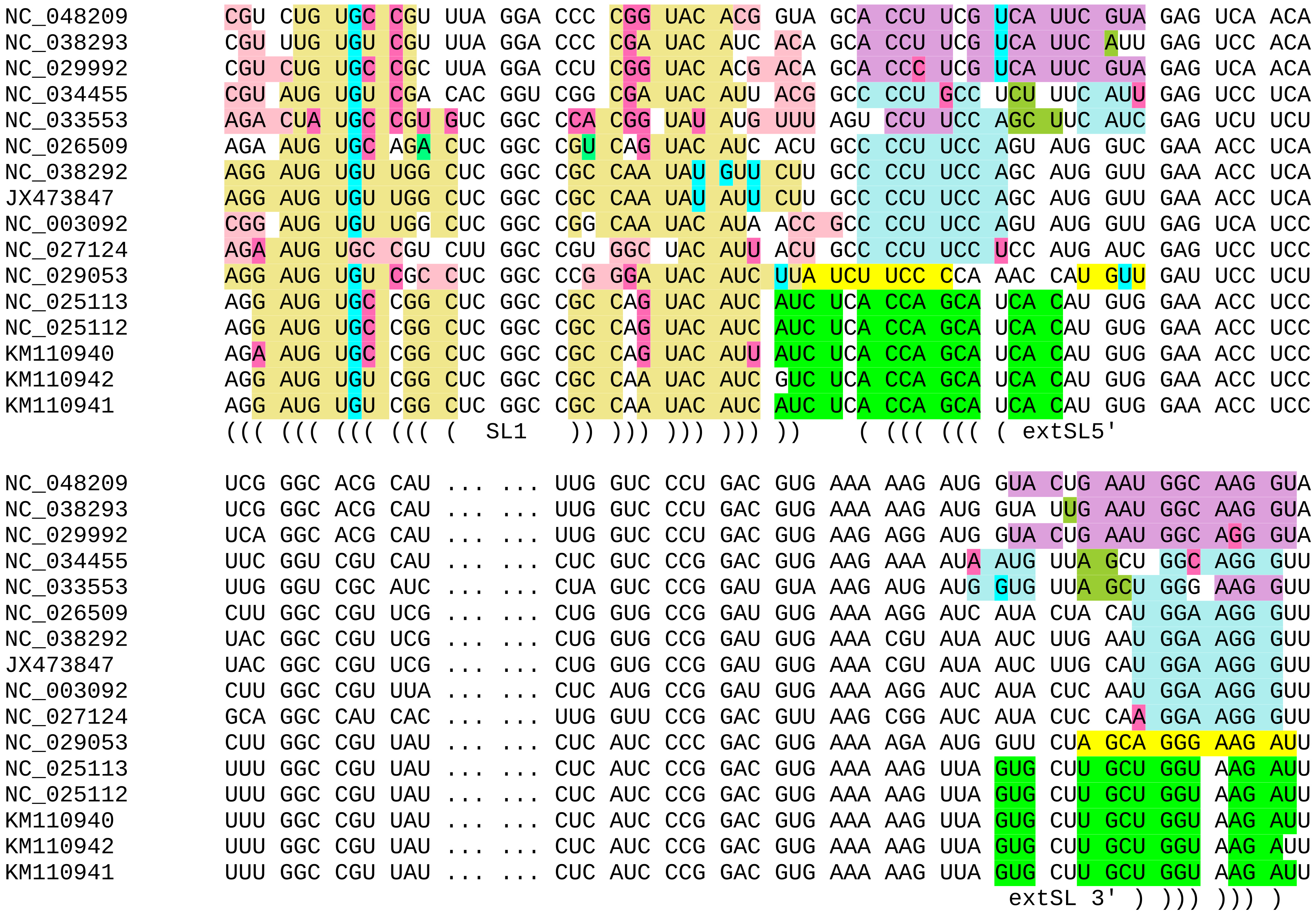

Supplement: Figure S3 — Potential conservation of SL1 and extSL in subfamily Simarterivirinae. [file jvi.02167-24-s0003.tif]

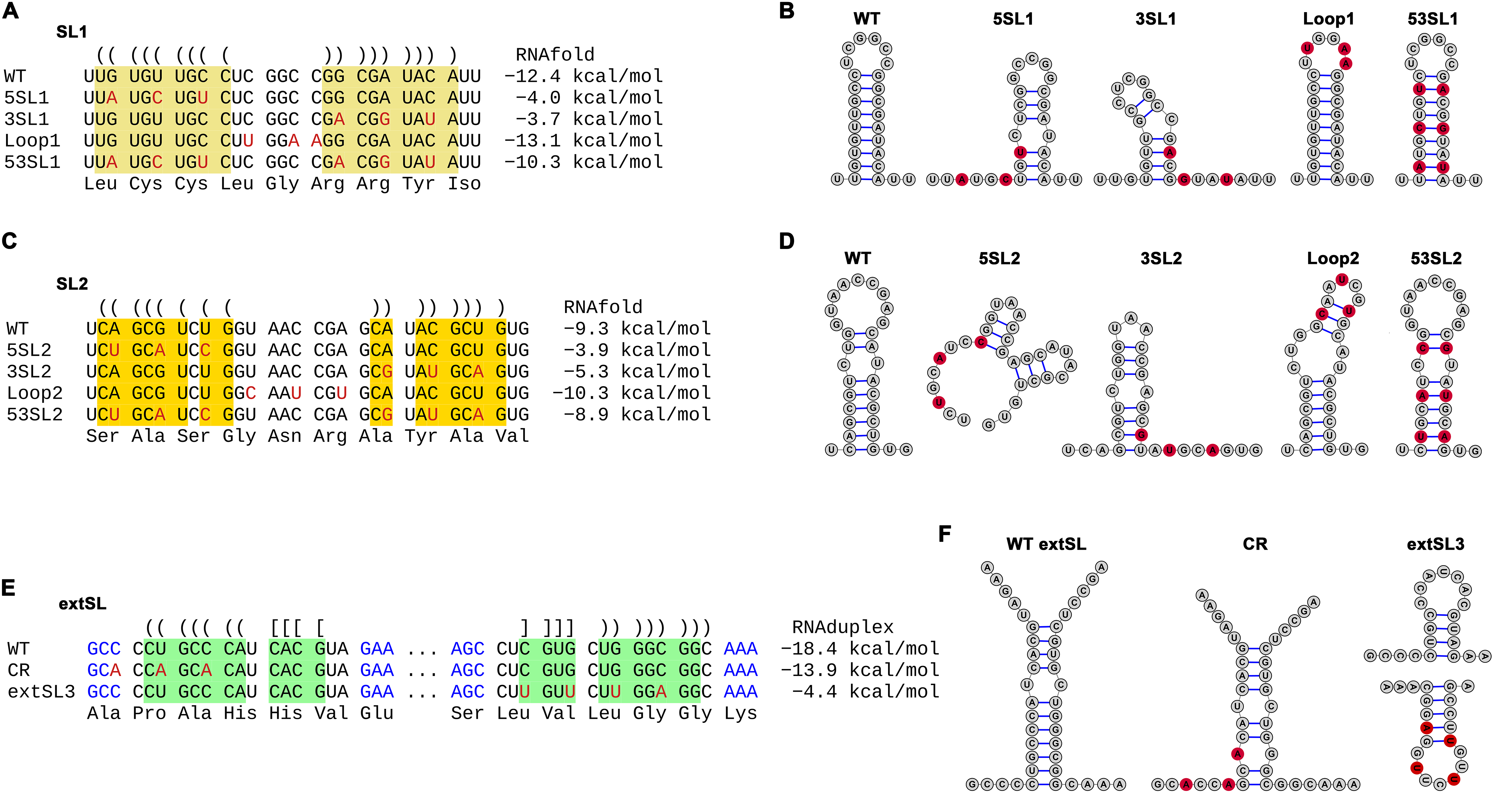

Supplement: Figure S4 — Predicted M ORF RNA structures containing mutations in the stem-loop region. [file jvi.02167-24-s0004.tif]

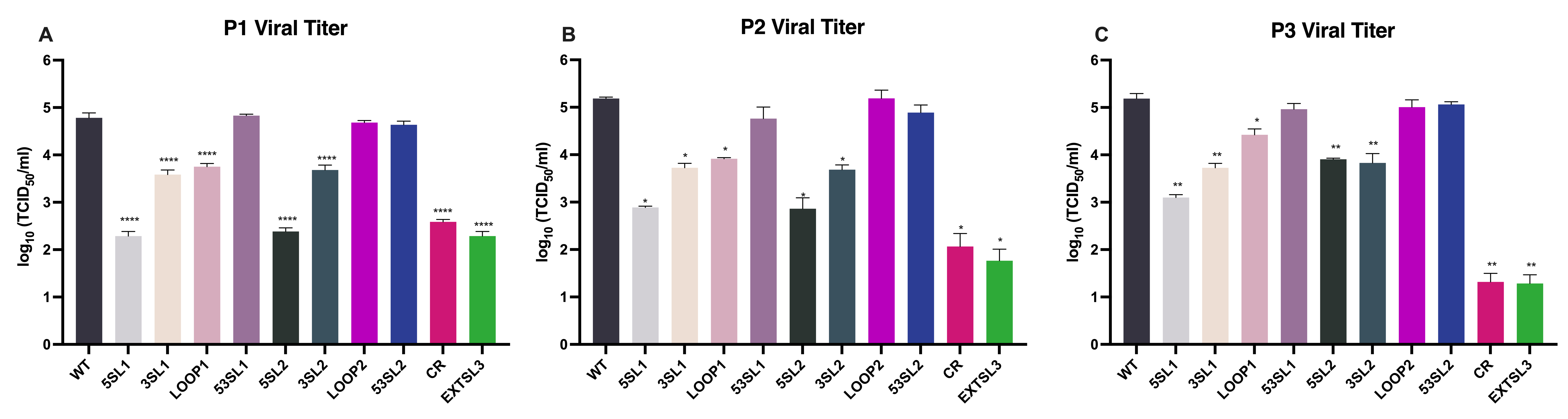

Supplement: Fig. S5 — Comparison of the viral titer of stem-loop mutants with wild-type virus in serial cell culture passages. [file jvi.02167-24-s0005.tiff]
