# Supplementary material for: An intra-family conserved high-order RNA structure within the M ORF is important for arterivirus subgenomic RNA accumulation and infectious virus production
Source: J Virol. 2025 Apr 7;99(5):e02167-24. doi: 10.1128/jvi.02167-24 (PMC7617654; doi:10.1128/jvi.02167-24)
Supplement: Table S1 — Stability of PRRSV stem-loop mutants in cell culture passage 3. [file jvi.02167-24-s0007.docx]

| **Constructs** | | **Mutation Design** | **Mutation Stability** | |  |  |  |  |
| --- | --- | --- | --- | --- | --- | --- | --- | --- |
| WT* | | Wild type | Wild type | |  |  |  |  |
| 5SL1 | WT | 14333- TTG TGT TGC CTC GGC CGG CGA TAC ATT -14359**^#^**  14333- TTA TGC TGT CTC GGC CGG CGA TAC ATT -14359 | Mutation maintained | |  |  |  |  |
|  | 5SL1 |  |  |  |  |  |  |  |
| 3SL1 | WT | 14333- TTG TGT TGC CTC GGC CGG CGA TAC ATT -14359  14333- TTG TGT TGC CTC GGC CGA CGG TAT ATT -14359 | Mutation maintained | |  |  |  |  |
|  | 3SL1 |  |  |  |  |  |  |  |
| Loop1 | WT | 14333- TTG TGT TGC CTC GGC CGG CGA TAC ATT -14359  14333- TTG TGT TGC CTT GGA AGG CGA TAC ATT -14359 | Revertant detected |  | |  |  |  |
|  | Loop1 |  |  |  |  |  |  |  |
| 53SL1 | WT | 14333- TTG TGT TGC CTC GGC CGG CGA TAC ATT -14359  14333- TTA TGC TGT CTC GGC CGA CGG TAT ATT -14359 | Mutation maintained | |  |  |  |  |
|  | 53SL1 |  |  |  |  |  |  |  |
| SL2 | WT | 14408- TCA GCG TCT GGT AAC CGA GCA TAC GCT GTG -14437  14408- TCT GCA TCC GGT AAC CGA GCA TAC GCT GTG -14437 | Revertant detected | | | |  | |
|  | 5SL2 |  |  |  |  |  |  |  |
| 3SL2 | WT | 14408- TCA GCG TCT GGT AAC CGA GCA TAC GCT GTG -14437  14408- TCA GCG TCT GGT AAC CGA GCG TAT GCA GTG -14437 | Mutation maintained | |  |  |  |  |
|  | 3SL2 |  |  |  |  |  |  |  |
| Loop2 | WT | 14408- TCA GCG TCT GGT AAC CGA GCA TAC GCT GTG -14437  14408- TCA GCG TCT GGC AAT CGT GCA TAC GCT GTG -14437 | Mutation maintained | |  |  |  |  |
|  | Loop2 |  |  |  |  |  |  |  |
| 53SL2 | WT | 14408- TCA GCG TCT GGT AAC CGA GCA TAC GCT GTG -14437  14408- TCT GCA TCC GGT AAC CGA GCG TAT GCA GTG -14437 | Mutation maintained | |  |  |  |  |
|  | 53SL2 |  |  |  |  |  |  |  |
| CR | WT | 14363- GCC CCT GCC -14371  14363- GCA CCA GCA -14371 | Mutation maintained | |  |  |  |  |
|  | CR |  |  |  |  |  |  |  |
| extSL3 | WT | 14366- CCT GCC CAT CAC GTA … CTC GTG CTG GGC GGC -14506  14366- CCT GCC CAT CAC GTA … CTT GTT CTT GGA GGC -14506 | Mutation maintained | |  |  |  |  |
|  | extSL3 |  |  |  |  |  |  |  |

*WT: wild type; # Numbers represent nucleotide position in the genome of PRRSV-1 isolate SD01-08 (GenBank accession number DQ489311.1).

**Table S1. Stability of PRRSV stem-loop mutants in cell culture passage 3**
